# Enhancing Broiler Production With Humic Acid and Probiotics: Effects on Growth Performance, Carcass Traits, Immune Response, Gut Microbiota, and Economic Feasibility

**DOI:** 10.1155/vmi/5944905

**Published:** 2026-08-05

**Authors:** Md. Khalaquzzman, Md. Mufazzal Hossain, Md. Imran Hossain

**Affiliations:** ^1^ Department of Animal Nutrition, Genetics and Breeding, Faculty of Animal Science and Veterinary Medicine, Sher-e-Bangla Agricultural University (SAU), Dhaka 1207, Bangladesh, sau.edu.bd

**Keywords:** broiler, carcass, growth performance, immune response, lymphocyte

## Abstract

**Background:**

Humic acid and probiotics are increasingly used as natural alternatives to antibiotic growth promoters in poultry production. Humic substances have been reported to support nutrient absorption and gut health, while probiotics enhance microbial balance and immune function.

**Objectives:**

This study aimed to evaluate the individual and combined effects of humic acid and probiotics on growth performance, carcass characteristics, immune parameters, caecal bacterial populations (*Escherichia coli*, *Salmonella* spp., and *Lactobacillus* spp.), and the financial feasibility of broiler production.

**Methods:**

A total of 195 one‐day‐old Lohmann Meat (Indian River) broiler chicks were allocated in a completely randomized design with five dietary treatments and 3 replicates of 13 birds each. Dietary treatments were as follows: a basal diet (control), a basal diet with 0.05% humic acid, a basal diet with 0.10% humic acid, a basal diet with 0.02% probiotics, and a basal diet containing both 0.05% humic acid and 0.02% probiotics.

**Results:**

Dietary inclusion of humic acid or probiotics did not significantly affect (*p* > 0.05) body weight gain, feed intake, or feed conversion ratio at 28 days (market age), but significant improvements were observed at 7, 14, and 21 days. However, the combined supplementation of 0.05% humic acid and 0.02% probiotics significantly improved several carcass traits (thigh, drumstick, back, wing, and liver weights), reduced caecal pathogenic bacteria (*E. coli* and *Salmonella spp*.), increased beneficial *Lactobacillus spp*., and enhanced immune parameters (higher lymphocyte percentage and lower white blood cell counts) compared to the control (*p* < 0.05). Hematological values remained within normal physiological ranges across all treatments. Economic analysis revealed that the combined supplementation group recorded the highest net present value (176.44 USD), internal rate of return (43.51%), and benefit–cost ratio (1.22), and the shortest payback period (1.93 years).

**Conclusions:**

Overall, this study demonstrated that dietary supplementation with humic acid and probiotics, particularly in combination, improved carcass traits, reduced pathogenic bacterial loads, enhanced selected immune parameters, and increased economic returns without affecting growth performance at 28 days.

## 1. Introduction

Antimicrobials have been used in poultry feed for over 5 decades to improve growth and prevent diseases. However, the excessive use of antibiotic growth promoters (AGPs) has led to the development of antimicrobial resistance, posing serious risks to human and animal health [1]. As a result, many countries have restricted the use of AGPs as feed additives for enhancing growth in poultry production and have stimulated the search for alternative strategies to replace antibiotics, including probiotics and prebiotics [2]. Therefore, identifying safe and efficient substitutes for AGPs that maintain broiler health and productivity remains a key challenge in poultry production.

The application of probiotics has attracted considerable attention as a promising strategy to stabilize gut microbial ecology, enhance nutrient utilization, and modulate immune responses in broilers through mechanisms such as competitive exclusion of pathogens, organic acid production, and improved intestinal barrier integrity [3–9]. In broiler nutrition, various probiotic species belonging to the genera *Lactobacillus*, *Streptococcus*, *Bacillus*, *Bifidobacterium*, *Enterococcus*, *Aspergillus*, *Candida*, and *Saccharomyces* are commonly utilized [10–12]. Probiotics alter the intestinal ecosystem through the production of digestive enzymes, reduction of intestinal pH, and enhancement of enzymatic activity within the gastrointestinal tract [13, 14]. Recent studies have reported that probiotic supplementation improves growth performance, carcass traits, and immune indices in broiler chickens, although responses vary depending on strain, dose, diet composition, and management conditions [15–18]. Among probiotics, *Bacillus* species are the most frequently used in poultry production due to their ability to form endospores, enabling survival under low pH, high bile salt concentration, and elevated temperature, as well as germination within the gastrointestinal tract [19, 20]. Supplementation with *B. subtilis* or *B. licheniformis* has been reported to promote beneficial gut bacteria and reduce pathogenic taxa, including *Salmonella* spp., in broilers [21–25].

In addition to probiotics, humic acid (HA) has emerged as a promising functional feed additive in poultry nutrition [26]. Humic substances are complex organic compounds formed during the decomposition of plant and microbial residues and are associated with diverse biological functions [27]. In broilers, dietary HA has been reported to reduce pathogenic bacteria such as *Escherichia coli*, *Salmonella* spp., and *Clostridium perfringens*; support intestinal integrity; and promote nutrient absorption and growth efficiency [28, 29]. The beneficial effects of humic substances are multifactorial and may involve toxin binding and chelation of harmful metabolites, modulation of intestinal pH [30], and improvements in intestinal morphology that support nutrient absorption and growth performance [31, 32]. In addition, HA has been reported to modulate immune responses through effects on humoral and cellular immunity [33]. For instance, the authors in [34] observed increased lymphocyte numbers in broilers supplemented with HA, potentially mediated by enhanced IL‐2 production and IL‐2 receptor expression. However, variations in HA composition, source, and dosage often result in inconsistent responses across studies [26]. Collectively, these properties suggest that HA can create a favorable intestinal environment that may enhance the effectiveness of probiotic supplementation in broiler chickens.

Although probiotics and HA have been extensively studied as alternatives to AGPs, most research has focused on their individual effects. Evidence on their combined supplementation remains limited. Therefore, this study was designed to evaluate the individual and combined effects of HA and probiotics on growth performance, carcass characteristics, hematological parameters, caecal bacterial populations (*E. coli, Salmonella* spp.*, and Lactobacillus spp*.), and economic returns in broilers. It was hypothesized that HA and probiotics, alone or combined, would improve growth performance, carcass traits, selected immune indices, caecal microbiota (*E. coli*, *Salmonella spp*., and *Lactobacillus spp*.) counts, and economic returns in broilers.

## 2. Materials and Methods

### 2.1. Birds, Design, and Experimental Diets

**FIGURE 1:**
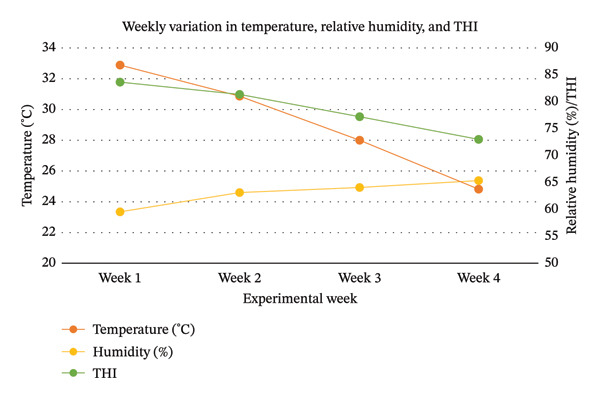
Weekly variation in temperature, relative humidity, and THI.

The experiment was conducted at the National Research and Development (RND) Poultry Farm, National Hatchery Ltd., Dighulia, Tangail, Bangladesh. A total of 195 one‐day‐old Lohmann Meat (Indian River) broiler chicks were used in a 28‐day trial. This commercial strain is widely used in Bangladesh and represents commonly adopted broiler genetics under local production systems. Day‐old chicks with an average initial body weight of 40 ± 0.5 g were procured from National Hatchery Ltd., Tangail, Bangladesh. Prior to chick placement, all pens, feeders, and drinkers were thoroughly cleaned and disinfected to ensure proper hygienic conditions throughout the experimental period. Chicks were individually weighed at placement and randomly allocated to five dietary treatments in a completely randomized design (CRD) with three replicates of 13 birds each to ensure uniform initial body weight distribution among treatments. The sample size and replication structure were selected based on previously published broiler nutrition studies conducted under comparable experimental conditions to ensure adequate statistical power and reliable detection of treatment effects [35–37]. Five isonitrogenous and isocaloric diets were formulated: (1) basal diet (control), (2) basal diet + 0.05% HA (HA0.05%), (3) basal diet + 0.10% HA (HA0.10%), (4) basal diet + 0.02% probiotics (Pro0.02%), and (5) basal diet + 0.05% HA + 0.02% probiotics (HA0.05% + Pro0.02%). The commercial HA product (Minerva, Pacta, Italy), composed of HA, fulvic acid (FA), and humin, was procured from Hoovers Agro Vet Ltd. (Bangladesh). The probiotic product (ENTEROSURE, Kemin Industries, India), containing dried *Bacillus subtilis* and *Bacillus licheniformis* fermentation products with a guaranteed total spore count of 6 × 10^8^ colony‐forming units (CFUs)/g, was obtained from Kemin Industries. The inclusion levels were selected based on previously reported effective dietary ranges (0.05%–0.10% for HA and approximately 0.02% for probiotics) and manufacturer recommendations. The starter diet was provided from Days 1 to 10, and the grower diet was offered from Days 11 to 28 of the experiment (Table 1). The starter diet was supplied in crumble form, while the grower diet was provided in pellet form. Birds were housed in floor pens (1.5 × 2.0 m; 3 m^2^) containing fresh rice husk litter as bedding material. The stocking density was 4.33 birds/m^2^. Each pen was equipped with manual feeders and drinkers. Feed and fresh drinking water were supplied ad libitum, and feed intake (FI) was recorded daily throughout the experimental period. The ambient temperature was maintained at approximately 33°C initially and gradually reduced by 2°C–3°C per week to reach approximately 24°C by the end of the trial. Average weekly relative humidity ranged from 60% to 65% throughout the experimental period. The temperature–humidity index (THI) was calculated according to the equation previously described by the authors in [38] as follows: THI = (0.8 × T) + (RH × (*T* − 14.4)/100) + 46.4, where *T* is the ambient temperature (°C) and RH is the relative humidity (%). Weekly temperature, relative humidity, and THI values during the experimental period are presented in Figure 1. A continuous lighting schedule (24 h light) was provided from Days 1 to 3, followed by 18 h light from Days 4 to 7, and then 23 h light until the end of the trial. Ventilation was controlled using exhaust fans and adjustable air inlets to ensure adequate air circulation and maintain suitable environmental conditions. Birds were monitored daily for general health status and welfare conditions throughout the study. The vaccination and medication program applied during the experiment is provided in Table 2. Strict biosecurity measures were maintained throughout the experimental period, with potassium permanganate footbaths maintained daily at the entrance and exit of the poultry house to minimize the risk of disease transmission. No AGPs or therapeutic antibiotics were administered during the experimental period.

**TABLE 1:** The ingredients and chemical composition of the basal diet (as‐fed basis, %).

Ingredients (%)	Starter (1–10 days)	Grower (11–28 days)
Corn	55.20	58.93
Soybean meal, 48%	29.70	25.11
Soybean oil	3.00	3.49
Rice bran	3.68	3.15
Poultry meal	3.80	5.30
Limestone	3.00	2.50
Dicalcium phosphate	0.25	0.25
Sodium bicarbonate	0.20	0.20
L‐Lysin HCl	0.15	0.15
DL‐methionine	0.20	0.20
Threonine	0.05	0.05
Common salt	0.40	0.30
Choline chloride, 60%	0.10	0.10
Vitamin mineral premix^1^	0.25	0.25
Phytase	0.02	0.02
Total	100.00	100.00
Chemical composition		
Metabolizable energy (Kcal/kg)	3001.00	3099.24
Crude protein (%)	21.99	20.94
Calcium (%)	1.03	0.85
Available phosphorous (%)	0.45	0.40
Lysine (%)	1.20	1.00
Methionine + cystine (%)	0.85	0.72
Threonine (%)	0.80	0.70

^1^Provides per kg of diet: vitamin A, 12,000 IU; vitamin D_3_, 2500 IU; vitamin E, 10 mg; vitamin K_3_, 2 mg; vitamin B_1_, 1 mg; vitamin B_2_, 5 mg; vitamin B_6_, 1.5 mg; vitamin B_12_, 10 μg; niacin, 25 mg; calcium pantothenate, 10 mg; folic acid, 1 mg; biotin, 50 μg; Mn, 60 mg; Zn, 50 mg; Fe, 30 mg; Cu, 5 mg; I, 1 mg; Se, 0.1 mg.

**TABLE 2:** Vaccination and supplementation schedule during the experimental period.

Age (day)	Disease/supplementation	Product	Composition	Dose	Route of administration	Administration period
Day 3	Newcastle disease + infectious bronchitis (ND + IB)	CEVAC BI L	Live NDV (Hitchner B1) + IBV (Massachusetts B48)	1 drop/bird	Eye drop	Single dose
Day 3–5	Supportive supplementation	Liva‐Vit Liquid	Liver extract + vitamin B complex	1 mL/1–2 L drinking water	Drinking water	3 consecutive days
Day 10–12	Supportive supplementation	Renasol AD_3_E	Vitamins A, D3, and E	1 mL/3–4 L drinking water	Drinking water	3 consecutive days
Day 11	Infectious bursal disease (IBD)	CEVA IBD L	Live IBDV (Winterfield 2512 G‐61 strain)	1 dose/bird	Drinking water	Single dose
Day 18	Infectious Bursal disease (IBD)	CEVAC IBD L	Live IBDV (Winterfield 2512 G‐61 strain)	1 dose/bird	Drinking water	Single dose
Day 21	Newcastle disease + infectious bronchitis (ND + IB)	CEVAC BI L	Live NDV (Hitchner B1) + IBV (Massachusetts B48)	1 dose/bird	Drinking water	Single dose

### 2.2. Growth Performance

Feed offered and leftover feed were measured daily to determine how much feed each bird consumed each week. The body weight of chicks was recorded at arrival and then weekly using a digital weighing scale. The feed conversion ratio (FCR) was calculated from the feed intake and body weight gain (BWG) values. Growth performance parameters, including average FI, BWG, and FCR, were calculated using the following formulas [12, 39, 40]:

(1) FCR=Total Feed Intake g Total Weight Gain g.

(2) Average FI g/bird=Total Feed Offered g −Feed Leftover gNumber of Birds,

(3) BWG g/bird=Final Body Weight g−Initial Body Weight g,

### 2.3. Carcass Yield

At the end of the experiment, three birds from each replicate (*n* = 9 birds per treatment) were randomly selected and slaughtered to evaluate carcass traits and internal organ weights. The hot carcass, heart, liver, spleen, gizzard, thigh, drumstick, back, and wings were individually weighed using a digital scale. The dressing percentage was calculated as the ratio of carcass weight to live (slaughter) weight, expressed as a percentage.

### 2.4. Immune Parameters

At the end of the experiment, blood samples were randomly collected from each replicate of every treatment group. Approximately 2 mL of blood was drawn aseptically from the wing vein using a sterile syringe and transferred into vacutainer tubes containing ethylenediaminetetraacetic acid (EDTA) as an anticoagulant to prevent coagulation. The samples were promptly transported to the laboratory and analyzed within a few hours of collection using an automated hematology analyzer. The immune and hematological parameters measured included hemoglobin (Hb) (g/dL), white blood cell (WBC) count (× 10^3^/μL), red blood cell (RBC) count × (10^6^/μL), platelet count (× 10^5^/μL), neutrophil (%), lymphocyte (%), monocyte (%), eosinophil (%), and packed cell volume (PCV) (%).

### 2.5. Microbial Population

Caecal contents were aseptically collected from the same slaughtered broilers for enumeration of *Escherichia coli*, *Salmonella spp*., and *Lactobacillus spp*. Fresh digesta samples were immediately transferred into sterile tubes for analysis. Enumeration of microbial populations followed the protocol of [41]. Homogenized samples were serially diluted (10^−1^ to 10^−7^) in sterile physiological saline, and 0.1 mL from each dilution was spread on Eosin Methylene Blue (EMB) agar, Salmonella–Shigella (SS) agar, and MRS agar (HiMedia, India) for bacterial isolation. The plates were incubated at 37°C for 24 h to enumerate *Escherichia coli*, *Salmonella spp*., and *Lactobacillus spp*. Visible microbial colonies were counted within the 30–300 CFU/plate range based on typical colony morphology immediately after incubation, and the results were expressed as log_10_ CFUs per gram of caecal content.

### 2.6. Economic Feasibility

#### 2.6.1. Net Present Value (NPV)

NPV is a financial indicator used to measure the present value of the net returns obtained from an investment. In broiler chicken production, the NPV can be calculated as follows [42]:

(4) NPV=∑t=inBt−Ct1+it−I0,

where *B*_*t*_ = benefits (revenue) in year *t*, *C*_*t*_ = costs in year *t*, *i* = discount rate, *n* = project age (years), and *I*_0_ = initial investment (year 0 cost).

#### 2.6.2. Internal Rate of Return (IRR)

The IRR is the discount rate that makes the NPV of a project equal to zero. According to [43], IRR is calculated by the formula as follows:

(5) IRR=i2+NPV1 NPV1−NPV2i2−i1,

where *i*_1_ = lower discount rate giving positive NPV, *i*_2_ = higher discount rate giving negative NPV, NPV_1_ = NPV at discount rate *i*_1_, and NPV_2_ =  NPV at discount rate *i*_2_.

#### 2.6.3. Benefit–Cost Ratio (BCR)

The BCR was computed as the ratio of the present value of total benefits to the present value of total costs. It helps to analyze the financial efficiency of the farms [44].

(6) BCR=PVBPVC,

where PV(B) = present value of total benefits (revenues) and PV(C) = present value of total costs (including fixed and variable costs).

#### 2.6.4. Payback Period

The payback period measures the time required for an investment to be recovered through the net cash inflows it generates. Mathematically, the payback period of the project was estimated by using the following formula:

(7) payback period=initial investmentannual net cash flow NCF .

Economic indicators were calculated assuming a 5‐year project lifespan and a 10% annual discount rate. Annual NCF was estimated based on five production cycles per year, accounting for the grow‐out period, interflock downtime, and seasonal production adjustments under local climatic conditions in Bangladesh and standard commercial management conditions. NPV, IRR, BCR, and payback period were derived from discounted annual cash flows following established agricultural investment evaluation procedures.

### 2.7. Statistical Analysis

Data were analyzed using the general linear model (GLM) procedure in IBM SPSS Statistics (Version 27.0) following a CRD. For growth performance and economic parameters, each pen was considered the experimental unit (*n* = 3 pens per treatment). For carcass traits, hematological parameters, caecal bacterial counts, and individual birds were considered the experimental unit (*n* = 9 birds per treatment; 3 birds per replicate). Data were subjected to one‐way analysis of variance (ANOVA), with dietary treatment (control, HA0.05%, HA0.10%, Pro0.02%, and HA0.05% + Pro0.02%) included as the fixed effect. The statistical model used was as follows:

(8) Yij=μ+Ti+εij,

where *Y*_*ij*_ is the observation for the *j*th replicate in the *i*th dietary treatment, μ is the overall mean, *T*_*i*_ is the fixed effect of dietary treatment, and *ε*_*ij*_ is the random error term.

Prior to ANOVA, data were tested for normality using the Shapiro–Wilk test and for homogeneity of variance using Levene’s test. When significant treatment effects were detected (*p* < 0.05), means were separated using Tukey’s multiple comparison test. Results are presented as means ± standard error of the mean (SEM).

## 3. Results

### 3.1. Growth Performance

The effects of dietary supplementation with HA and/or probiotics on broiler growth performance and FCR are summarized in Table 3, as evaluated by BWG, FI, and FCR at different time points. The BWG at 7 days of age differed significantly among treatments, with the highest values observed in HA0.05% + Pro0.02% group (141.67 ± 1.45 g), representing a 1.68% increase compared to the control group (139.33 ± 2.03 g), whereas the lowest BWG was recorded in Pro0.02% (119.33 ± 4.98 g), corresponding to a 14.36% decrease relative to the control (*p* = 0.0136). However, FI and FCR at 7 days did not differ significantly among treatments (*p* = 0.8253 and *p* = 0.0698, respectively). The BWG at 14 days of age, differed significantly (*p* = 0.0327), with the highest BWG recorded in HA0.10% (415.67 ± 11.39 g) and HA0.05% + Pro0.02% (420.00 ± 5.00 g), representing an 18.64% increase compared to the control group (354.00 ± 11.37 g). FI also varied among treatments at this age (*p* < 0.01), with the highest intake recorded in the combined HA–probiotic group (543.33 ± 0.67 g). In contrast, FCR at 14 days did not differ significantly (*p* = 0.0633). Broilers receiving dietary probiotics and/or HA showed significant differences in BWG at 21 days of age (*p* = 0.0353), with the highest values observed in the HA0.05% + Pro0.02% (975.00 ± 28.87 g) groups, showing a 4.95% increase in BWG compared to the control (929.00 ± 23.64 g). A lower FCR was also found in the HA0.05% + Pro0.02% group (1.18 ± 0.04) at 21 days (*p* = 0.0305). In contrast, FI at 21 days did not differ among treatments (1138.67 ± 13.31 g in HA0.05% to 1174.67 ± 35.64 g in HA0.10%; *p* = 0.4508). By 28 days of age, BWG and FI did not differ significantly among treatments (*p* = 0.3917 and *p* = 0.3155, respectively), although the HA0.05% + Pro0.02% group showed numerically higher BWG (1660.67 ± 64.05 g). Similarly, no significant differences were observed in FCR at 28 days (*p* = 0.5622).

**TABLE 3:** Effects of humic acid and probiotics on body weight gain, feed intake, and feed conversion ratio.

Parameters	Treatments (mean ± SE)					SEM	*p* value			
	Con	HA0.05%	HA0.10%	Pro0.02%	HA0.05% + Pro0.02%		ANOVA	Linear	Quadratic	Cubic
BWG (g) at 7 days	139.33 ± 2.03^a^	132.00 ± 5.13^ab^	126.00 ± 4.62^b^	119.33 ± 4.98^b^	141.67 ± 1.45^a^	3.97	0.0136	0.5379	0.0027	0.0519
FI(g) at 7 days	174.00 ± 0.58	172.00 ± 2.52	170.67 ± 5.46	171.33 ± 1.86	169.33 ± 0.67	2.84	0.8253	0.2916	0.8545	0.7183
FCR at 7 days	1.25 ± 0.02	1.31 ± 0.07	1.36 ± 0.09	1.44 ± 0.05	1.20 ± 0.01	0.05	0.0698	0.8933	0.0175	0.095
BWG (g) at 14 days	354.00 ± 11.37^b^	396.67 ± 24.58^ab^	415.67 ± 11.39^a^	360.33 ± 16.50^b^	420.00 ± 5.00^a^	15.23	0.0327	0.0751	0.4953	0.0164
FI(g) at 14 days	526.00 ± 1.15^b^	505.67 ± 2.84^c^	529.00 ± 3.21^b^	519.00 ± 6.67^b^	543.33 ± 0.67^a^	3.59	< 0.01	0.0018	0.0019	0.4306
FCR at 14 days	1.49 ± 0.05	1.29 ± 0.09	1.27 ± 0.03	1.45 ± 0.07	1.29 ± 0.02	0.06	0.0633	0.2345	0.2136	0.0169
BWG (g) at 21 days	929.00 ± 23.64^ab^	933.00 ± 12.66^ab^	947.67 ± 13.22^a^	872.00 ± 8.54^b^	975.00 ± 28.87^a^	18.98	0.0353	0.6167	0.1604	0.0188
FI(g) at 21 days	1153.67 ± 29.87	1138.67 ± 13.31	1174.67 ± 35.64	1167.00 ± 25.35	1150.00 ± 6.55	14.20	0.4508	0.6501	0.3908	0.2089
FCR at 21 days	1.24 ± 0.02^b^	1.22 ± 0.01^b^	1.24 ± 0.03^b^	1.34 ± 0.03^a^	1.18 ± 0.04^b^	0.03	0.0305	0.9594	0.1023	< 0.01
BWG (g) at 28 days	1539.33 ± 40.54	1563.67 ± 50.76	1582.00 ± 29.77	1570.67 ± 8.09	1660.67 ± 64.05	43.07	0.3917	0.0967	0.5422	0.4489
FI(g) at 28 days	2155.33 ± 14.31	2121.67 ± 16.90	2145.33 ± 21.36	2159.33 ± 6.33	2162.33 ± 3.48	14.14	0.3155	0.2746	0.2564	0.1574
FCR at 28 days	1.40 ± 0.05	1.36 ± 0.05	1.36 ± 0.02	1.37 ± 0.01	1.30 ± 0.05	0.03	0.5622	0.1869	0.8373	0.3408

*Note:* Values are presented as mean ± standard error (SE). Means within a row bearing different superscripts differ significantly (*p* < 0.05), whereas rows without superscripts indicate no significant differences among treatments. HA0.05% and HA0.10% indicate inclusion of humic acid at 0.05% (0.5 g/kg) and 0.10% (1.0 g/kg) of feed, respectively. Pro0.02% indicates inclusion of probiotic at 0.02% (0.2 g/kg) of feed. Con = control.

Abbreviation: SEM, standard error of the mean.

### 3.2. Carcass Traits

The effects of HA and/or probiotics on carcass traits of broilers at the end of the experiment are shown in Table 4. The evaluated parameters included dressing percentage, major cut‐up parts (thigh, drumstick, back, and wing), and internal organs (liver, heart, gizzard, and spleen) across the five treatment groups. Dressing percentage did not differ significantly among treatments, although numerically higher values were observed in the HA0.10% (64.49 ± 0.13%) and HA0.05% + Pro0.02% (64.51 ± 0.20%) groups (*p* = 0.0588). The highest thigh weight was observed in the HA0.05% + Pro0.02% group (173.00 ± 5.77 g), which represented a 37.66% increase compared to the control group (125.67 ± 2.33 g) (*p* < 0.001). Drumstick weight also varied significantly (*p* = 0.0233), with the highest value observed in the HA0.05% + Pro0.02% group (150.33 ± 3.53 g), representing a 28.85% increase compared to the control group (116.67 ± 2.73 g), followed by HA0.10% (146.67 ± 2.33 g), Pro0.02% (135.00 ± 12.66 g), and HA0.05% (129.67 ± 3.84 g). Back weight showed a similar pattern, with higher back weights recorded in the HA0.10% (188.33 ± 6.39 g) and HA0.05% + Pro0.02% (190.33 ± 7.13 g) groups compared to the control (166.00 ± 5.86 g) (*p* = 0.0162). Wing weight differed significantly among treatments (*p* < 0.001), with the highest values observed in the HA0.05% + Pro0.02% group (87.33 ± 4.10 g), representing a 48.85% increase compared to the control group (58.67 ± 1.20 g). Regarding internal organs, liver weight differed significantly (*p* = 0.0286), with the highest value in the HA0.05% + Pro0.02% group (48.33 ± 1.85 g). Heart weight did not differ among treatments (*p* = 0.2915). Gizzard weight was statistically similar across all groups (*p* = 0.1735). Spleen weight showed no significant differences (*p* = 0.1522), although numerically higher values were observed in the supplemented groups.

**TABLE 4:** Effects of humic acid and probiotics on carcass traits of broiler at the end of the experiment.

Parameters	Treatments (mean ± SE)					SEM	*p* value			
	Con	HA0.05%	HA0.10%	Pro0.02%	HA0.05% + Pro0.02%		ANOVA	Linear	Quadratic	Cubic
Dressing (%)	62.85 ± 0.79	64.16 ± 0.14	64.49 ± 0.13	63.52 ± 0.30	64.51 ± 0.20	0.396	0.0588	0.0574	0.2210	0.0396
Thigh (g)	125.67 ± 2.33^c^	136.00 ± 1.00^bc^	149.00 ± 2.31^b^	135.67 ± 7.31^bc^	173.00 ± 5.77^a^	4.44	< 0.001	< 0.001	0.1268	0.0066
Drumstick (g)	116.67 ± 2.73^b^	129.67 ± 3.84^ab^	146.67 ± 2.33^a^	135.00 ± 12.66^ab^	150.33 ± 3.53^a^	6.33	0.0233	0.0046	0.3349	0.2774
Back (g)	166.00 ± 5.86^b^	164.67 ± 2.60^b^	188.33 ± 6.39^a^	175.33 ± 2.90^ab^	190.33 ± 7.13^a^	5.31	0.0162	0.0054	0.8446	0.8618
Wing (g)	58.67 ± 1.20^c^	64.33 ± 2.33^c^	74.00 ± 0.58^b^	62.00 ± 1.53^c^	87.33 ± 4.10^a^	2.30	< 0.001	< 0.001	0.0667	< 0.001
Liver (g)	42.33 ± 0.88^b^	42.67 ± 0.88^b^	44.67 ± 1.45^ab^	41.67 ± 1.20^b^	48.33 ± 1.85^a^	1.31	0.0286	0.0239	0.1483	0.0819
Heart (g)	8.33 ± 0.33	9.33 ± 0.33	9.67 ± 0.67	9.33 ± 0.33	9.33 ± 0.33	0.42	0.2915	0.1645	0.1218	0.4705
Gizzard	22.67 ± 1.20	28.00 ± 0.58	26.33 ± 2.40	26.67 ± 1.67	29.00 ± 2.08	1.71	0.1735	0.0629	0.5465	0.1275
Spleen (g)	2.33 ± 0.33	3.00 ± 0.01	3.33 ± 0.33	3.33 ± 0.33	3.33 ± 0.33	0.30	0.1522	0.0328	0.1660	0.7310

*Note:* Values are presented as mean ± standard error (SE). Means within a row bearing different superscripts differ significantly (*p* < 0.05), whereas rows without superscripts indicate no significant differences among treatments. HA0.05% and HA0.10% indicate inclusion of humic acid at 0.05% (0.5 g/kg) and 0.10% (1.0 g/kg) of feed, respectively. Pro0.02% indicates inclusion of probiotic at 0.02% (0.2 g/kg) of feed. Con = control.

Abbreviation: SEM, standard error of the mean.

### 3.3. Immune Parameters

The effects of dietary HA and/or probiotics on immune parameters of broiler chickens are presented in Table 5. The evaluated hematological parameters included Hb, WBCs, RBCs, platelets, and differential leukocyte counts (neutrophils, lymphocytes, monocytes, and eosinophils), as well as PCV. Hb concentrations did not differ significantly among treatments (*p* = 0.5007), with the lowest level observed in Pro0.02% (8.37 ± 0.47 g/dL) and the highest in HA0.05% (9.69 ± 0.48 g/dL). Similarly, RBC, platelet counts, monocytes, eosinophils, and PCV showed no significant variation among treatments (*p* > 0.05). In contrast, WBC counts exhibited significant differences (*p* = 0.0282), with the lowest value recorded in the HA0.05% + Pro0.02% group (7.62 ± 0.52 × 10^3^/μL) representing a 52.14% reduction compared to the highest value recorded in the HA0.05% group (15.92 ± 2.02 × 10^3^/μL). Neutrophil percentages also differed significantly (*p* = 0.0486), with increased levels in HA0.10% (81.67 ± 1.20%) compared to the control (74.00 ± 3.21%) and reduced proportions in Pro0.02% (70.33 ± 4.09%) and HA0.05% + Pro0.02% (69.67 ± 1.45%). Lymphocyte counts showed significant differences (*p* = 0.0388), with the HA0.05% + Pro0.02% group (27.67 ± 2.40%) exhibiting an 80.50% increase compared to the HA0.10% group (15.33 ± 0.33%).

**TABLE 5:** The effect of humic acid and probiotics on immune parameters.

Parameters	Treatments (mean ± SE)					SEM	*p* value			
	Con	HA0.05%	HA0.10%	Pro0.02%	HA0.05% + Pro0.02%		ANOVA	Linear	Quadratic	Cubic
Hemoglobin (g/dL)	9.64 ± 0.96	9.69 ± 0.48	9.55 ± 0.91	8.37 ± 0.47	8.47 ± 0.52	0.70	0.5007	0.1293	0.7286	0.5221
WBC (× 10^3^/μL)	13.90 ± 0.95^a^	15.92 ± 2.02^a^	13.52 ± 1.54^a^	10.86 ± 2.09^ab^	7.62 ± 0.52^b^	1.55	0.0282	0.0048	0.0922	0.4514
RBC (× 10^6^/μL)	3.80 ± 0.83	4.38 ± 0.98	3.25 ± 0.50	2.80 ± 0.58	2.60 ± 0.49	0.70	0.4139	0.1030	0.7433	0.3969
Platelet (× 10^5^/μL)	2.25 ± 0.48	2.71 ± 0.58	3.60 ± 0.62	2.39 ± 0.75	2.35 ± 0.73	0.64	0.5805	0.9526	0.2227	0.7261
Neutrophil (%)	74.00 ± 3.21^ab^	73.33 ± 1.33^ab^	81.67 ± 1.20^a^	70.33 ± 4.09^b^	69.67 ± 1.45^b^	2.55	0.0486	0.1781	0.0660	0.8402
Lymphocyte (%)	19.67 ± 3.18^ab^	20.33 ± 1.45^ab^	15.33 ± 0.33^b^	24.00 ± 3.21^a^	27.67 ± 2.40^a^	2.39	0.0388	0.0261	0.0521	0.9313
Monocyte (%)	2.33 ± 0.88	2.67 ± 0.33	1.67 ± 0.67	2.00 ± 0.58	2.00 ± 0.58	0.63	0.8308	0.5201	0.7839	0.6279
Eosinophil (%)	4.00 ± 1.00	3.67 ± 0.88	4.67 ± 1.20	3.00 ± 0.01	2.67 ± 0.33	0.82	0.4749	0.2258	0.4032	1.000
PCV (%)	30.55 ± 5.78	38.59 ± 3.17	28.25 ± 2.21	25.74 ± 1.94	27.17 ± 2.15	3.37	0.1333	0.0957	0.6778	0.0628

*Note:* Values are presented as mean ± standard error (SE). Means within a row bearing different superscripts differ significantly (*p* < 0.05), whereas rows without superscripts indicate no significant differences among treatments. HA0.05% and HA0.10% indicate inclusion of humic acid at 0.05% (0.5 g/kg) and 0.10% (1.0 g/kg) of feed, respectively. Pro0.02% indicates inclusion of probiotic at 0.02% (0.2 g/kg) of feed. Con = control.

Abbreviation: SEM, standard error of the mean.

### 3.4. Caecal Microbial Populations

The effects of dietary HA and/or probiotics on the caecal microbial population of broiler chickens are presented in Table 6. Caecal *E. coli* counts differed significantly among treatments (*p* = 0.0027). The Pro0.02% group showed a 14.42% reduction in *E. coli* counts compared to the control group. The highest *E. coli* counts were observed in the control group (7.21 ± 0.00 log_10_ cfu/g), followed by HA0.05% (7.03 ± 0.09 log_10_ cfu/g), HA0.10% (6.63 ± 0.01 log_10_ cfu/g), and HA0.05% + Pro0.02% (6.24 ± 0.34 log_10_ cfu/g) groups, whereas significantly lower *E. coli* counts were recorded in Pro0.02% (6.17 ± 0.03 log_10_ cfu/g), indicating a strong inhibitory effect of probiotic supplementation. A similar pattern was observed for *Salmonella* spp., which differed significantly among treatments (*p* < 0.001). The highest count was recorded in the control group (7.39 ± 0.01 log_10_ cfu/g), followed by HA0.05% (6.82 ± 0.29 log_10_ cfu/g), HA0.10% (5.99 ± 0.34 log_10_ cfu/g), and Pro0.02% (5.87 ± 0.00 log_10_ cfu/g), while the lowest count was recorded in the HA0.05% + Pro0.02% group (4.71 ± 0.01 log_10_ cfu/g), which exhibited a 36.27% reduction in *Salmonella spp*. In contrast, caecal *Lactobacillus* populations increased significantly among treatments (*p* < 0.001). The lowest *Lactobacillus* count was observed in the control group (7.20 ± 0.00 log_10_ cfu/g), whereas higher values were recorded in HA0.05% (7.46 ± 0.00 log_10_ cfu/g), HA0.10% (7.44 ± 0.00 log_10_ cfu/g), and Pro0.02% (7.45 ± 0.00 log_10_ cfu/g) groups. The highest *Lactobacillus* population was observed in the combined HA0.05% + Pro0.02% treatment (7.52 ± 0.01 log_10_ cfu/g), which showed a 4.44% increase in *Lactobacillus* population compared to the control group (*p* < 0.001). This indicates that dietary supplementation with HA and probiotics promoted the proliferation of beneficial intestinal bacteria. Flock uniformity was not significantly affected by dietary treatments (*p* = 0.5846), with values ranging from 68.67 ± 8.67% in HA0.10% to 82.00 ± 5.86% in HA0.05%. Although HA supplemented groups showed numerically higher uniformity, the differences were not statistically significant.

**TABLE 6:** Effects of humic acid and probiotics on caecal microbial population and flock uniformity.

Parameters	Treatments (mean ± SE)					SEM	*P* value			
	Con	HA0.05%	HA0.10%	Pro0.02%	HA0.05% + Pro0.02%		ANOVA	Linear	Quadratic	Cubic
*E. coli* (log10 cfu/g)	7.21 ± 0.00^a^	7.03 ± 0.09^ab^	6.63 ± 0.01^bc^	6.17 ± 0.03^c^	6.24 ± 0.34^c^	0.16	0.0027	< 0.001	0.4750	0.1615
*Salmonella* (log10 cfu/g)	7.39 ± 0.01^a^	6.82 ± 0.29^a^	5.99 ± 0.34^b^	5.87 ± 0.00^b^	4.71 ± 0.01^c^	0.20	< 0.001	< 0.001	0.5547	0.2448
*Lactobacillus* (log10 cfu/g)	7.20 ± 0.00^c^	7.46 ± 0.00^b^	7.44 ± 0.00^b^	7.45 ± 0.00^b^	7.52 ± 0.01^a^	0.01	< 0.001	< 0.001	< 0.001	< 0.001
Flock uniformity	73.00 ± 10.15	82.00 ± 5.86	68.67 ± 8.67	69.67 ± 4.91	80.00 ± 3.06	7.01	0.5846	0.9416	0.5317	0.1838

*Note:* Values are presented as mean ± standard error (SE). Means within a row bearing different superscripts differ significantly (*p* < 0.05), whereas rows without superscripts indicate no significant differences among treatments. HA0.05% and HA0.10% indicate inclusion of humic acid at 0.05% (0.5 g/kg) and 0.10% (1.0 g/kg) of feed, respectively. Pro0.02% indicates inclusion of probiotic at 0.02% (0.2 g/kg) of feed. Con = control.

Abbreviation: SEM, standard error of the mean.

### 3.5. Economic Feasibility

The economic feasibility results of broiler production under different levels of HA and/or probiotics are presented in Table 7. The evaluated economic indicators included NPV, IRR, BCR, and payback period across the five treatment groups. NPV varied widely among treatments, with the highest value observed in the HA0.05% + Pro0.02% group (176.44 USD), followed by HA0.10% (93.93 USD) and HA0.05% (84.47 USD), whereas the lowest NPV was obtained in the control (45.33 USD). A similar pattern was reflected in IRR, which peaked in the HA0.05% + Pro0.02% group (43.51%), compared to 28.74% in HA0.10%, 27.15% in HA0.05%, and 19.40% in the control. The BCR values ranged from 1.14 in the control to 1.22 in the HA0.05% + Pro0.02% group, indicating improved economic efficiency with supplementation. Payback period followed an inverse trend, with the shortest recovery time recorded in the HA0.05% + Pro0.02% group (1.93 years), compared to 2.50–2.64 years in the single‐supplement groups and 3.04 years in the control.

**TABLE 7:** Total cost, return, and financial feasibility metrics for broilers fed humic acid and probiotic diets.

Parameters	Cost (in USD)				
	Con	HA0.05%	HA0.10%	Pro0.02%	HA0.05% + Pro0.02%
Fixed capital					
1. Housing	166.67	166.67	166.67	166.67	166.67
2. Equipment	11.25	11.25	11.25	11.25	11.25
3. Miscellaneous	5.17	5.17	5.17	5.17	5.17
Total investment	183.09	183.09	183.09	183.09	183.09
Variable cost					
1. Feed	44.83	44.13	44.62	44.62	44.91
2. Day old chick	19.50	19.50	19.50	19.50	19.50
3. Litter	4.58	4.58	4.58	4.58	4.58
4. Vaccine	2.33	2.33	2.33	2.33	2.33
5. Medicine	1.33	1.33	1.33	1.33	1.33
6. Electricity, labor, transportation, etc.	2.92	2.92	2.92	2.92	2.92
7. HA and probiotic cost	0	0.14	0.28	0.35	0.49
A. Total variable cost	75.49	74.93	75.56	75.63	76.06
Fixed cost					
1. Housing (5%)	8.33	8.33	8.33	8.33	8.33
2. Equipment and miscellaneous (10%)	1.64	1.64	1.64	1.64	1.64
B. Total fixed cost	9.97	9.97	9.97	9.97	9.97
Total cost (A + B)	85.46	84.90	85.53	85.60	86.03
Return	97.52	99.03	100.16	99.46	105.02
Net present value (NPV)	45.33	84.47	93.93	79.30	176.44
Internal rate of return (IRR)	19.40%	27.15%	28.74%	26.22%	43.51%
Benefit–cost ratio (BCR)	1.14	1.17	1.17	1.16	1.22
Payback period (years)	3.04	2.59	2.50	2.64	1.93

*Note:* HA0.05% and HA0.10% indicate inclusion of humic acid at 0.05% (0.5 g/kg) and 0.10% (1.0 g/kg) of feed, respectively. Pro0.02% indicates inclusion of probiotic at 0.02% (0.2 g/kg) of feed. Con = control.

## 4. Discussion

Although significant differences in BWG and FCR were observed during the early growth phases (7–21 days), the absence of significant differences at 28 days indicates that dietary HA and probiotics exerted the greatest influence during the early developmental period of broilers rather than throughout the entire production cycle. The early‐phase improvements may be attributed to the critical period of intestinal development and microbial colonization during the first 3 weeks of life, a stage at which broilers are particularly responsive to dietary modulation. Probiotics are known to enhance early gut microbial balance, stimulate digestive enzyme secretion, and reduce intestinal inflammation [4, 24, 45, 46], while HA may improve mineral utilization and gut integrity through its chelating and antimicrobial properties [47, 48]. These combined effects could have contributed to the improved nutrient absorption and growth observed during the starter phase. The lack of significant differences at 28 days may reflect compensatory growth in the control group, natural stabilization of the intestinal microbiota over time, or the relatively low inclusion levels used in this study. In addition, the modest sample size may have limited the statistical power to detect small but biologically relevant differences at the final time point. Although the combined HA0.05% + Pro0.02% group showed numerically higher BWG at 28 days, formal statistical interaction analysis was not conducted; therefore, the observed response should be interpreted as a potential additive effect rather than confirmed synergism. The present findings are consistent with previous studies reporting limited or inconsistent effects of low dietary inclusion levels of humic substances or probiotics on final body weight and FCR [49–53]. However, other authors have demonstrated improved FCR at higher inclusion levels of HA [54], suggesting that dose, product composition, environmental conditions, and bird genotype may influence the magnitude of response. Future studies incorporating gut morphology, nutrient digestibility measurements, and molecular markers of intestinal function would help clarify the mechanisms underlying the phase‐dependent effects observed in this study.

The observed differences in thigh, drumstick, back, wing, and liver weights indicate that the inclusion of HA and probiotics positively influenced carcass development in broiler chickens. Improved carcass traits may be associated with enhanced intestinal health and digestive efficiency, which can increase the availability of nutrients required for muscle development. Humic substances are reported to enhance digestive enzyme activity and improve intestinal morphology, thereby facilitating superior nutrient absorption and muscle accretion [52, 55]. Similarly, probiotics have been shown to increase protein digestion and maintain intestinal integrity, which collectively contribute to improved carcass traits [6, 56]. The increased thigh and drumstick weights in the combined HA0.05% + Pro0.02% group suggest a combined additive effect, possibly due to complementary roles of HA and probiotics in nutrient assimilation and gut microbial equilibrium. Despite the improvement in individual cut‐up parts, dressing percentage did not differ significantly among treatments. This may be attributed to proportional increases in both live body weight and carcass weight, thereby maintaining a relatively stable carcass‐to‐live weight ratio. Similar findings have been reported by the authors in [50], who observed that humic substances exerted limited influence on overall dressing percentage despite changes in specific carcass components.

The relatively stable hematological profile observed in the present study indicates that dietary supplementation with HA and probiotics did not adversely affect the physiological condition of broiler chickens. Similar findings were reported by the authors in [50, 52], who observed no significant alterations in hematological parameters following humic substance supplementation. Importantly, all measured values remained within the normal physiological ranges reported for broilers, indicating the safety of the dietary treatments. Although Hb, RBC, and PCV were unaffected, the increased lymphocyte percentage observed in the Pro0.02% group may suggest enhanced adaptive immune responsiveness. Probiotics have been reported to modulate immune function by stimulating lymphocyte proliferation and improving gut‐associated immune activity [15, 24, 57], which is consistent with the present findings. The reduced total WBC count in the combined HA0.05% + Pro0.02% group may reflect decreased systemic immune activation, potentially associated with improved microbial balance and reduced pathogenic bacterial load observed in the cecum. However, as functional immune assays such as antibody titers or cytokine profiling were not performed, definitive conclusions regarding immune modulation cannot be drawn. The increased neutrophil percentage observed in the HA0.10% group, consistent with the observations of [58], may indicate modulation of the innate immune response; nevertheless, without additional inflammatory or stress markers, this finding should be interpreted cautiously. Further studies incorporating comprehensive immunological assessments would be necessary to clarify the specific mechanisms underlying the hematological responses observed in this study.

Supplementation with HA and/or probiotics significantly decreased caecal *E. coli* and *Salmonella spp*. counts while increasing beneficial *Lactobacillus* populations, suggesting improved intestinal microbial balance. The antimicrobial effects of HA may be associated with its ability to bind toxins, alter intestinal pH, and interfere with pathogen colonization, while probiotics may suppress pathogenic bacteria through competitive exclusion, organic acid production, and enhancement of intestinal microbial stability. The greater reduction in pathogenic bacteria observed in the combined supplementation group suggests that HA and probiotics may exert complementary antimicrobial actions within the gastrointestinal tract. Similar reductions in *E. coli* following humic substance supplementation have been reported by the authors in [59, 60], while probiotic supplementation has been shown to suppress *Salmonella* spp. through competitive exclusion and microbial antagonism [61]. It is important to acknowledge that only two pathogenic bacterial groups and one beneficial bacterial group were enumerated in this study, and comprehensive microbiota profiling (e.g., 16S rRNA sequencing) was not performed. Therefore, while reductions in *E. coli* and *Salmonella spp*. indicate improved microbial control, broader conclusions regarding overall microbial community structure and other beneficial bacterial populations cannot be drawn. Future studies employing molecular microbiome analyses would provide a more comprehensive understanding of how combined HA and probiotic supplementation influences gut microbial ecology.

The economic analysis demonstrated that dietary supplementation with HA and probiotics improved key financial indicators of broiler production. All supplemented groups exhibited higher NPV, IRR, and BCR, along with a shorter payback period compared to the control group, with the greatest economic advantage observed in the HA0.05% + Pro0.02% treatment. The higher BCR values (> 1.0) across supplemented groups indicate favorable returns relative to investment costs, while the elevated IRR suggests improved profitability potential under the specified production conditions. The higher NPV, IRR, and BCR values observed in the combined supplementation group may be associated with improved carcass development and reduced pathogenic load, without substantial increases in production cost. However, economic performance in poultry production is strongly influenced by feed ingredient prices, market value of broilers, management conditions, and the discount rate assumptions applied in the analysis. Therefore, while the present findings suggest that combined supplementation may enhance economic efficiency under the specified conditions, further evaluation in diverse commercial production systems is required to confirm its broader financial applicability.

## 5. Conclusions

In conclusion, dietary supplementation with HA and probiotics, particularly the combination of HA0.05% + Pro0.02%, improved early growth performance, selected carcass traits, caecal microbial balance (reduced *E. coli* and *Salmonella spp*., and increased *Lactobacillus* spp.), and economic efficiency in broiler chickens. Although BWG at 28 days and most hematological parameters did not differ significantly among treatments, the supplements did not adversely affect the physiological condition of the birds. These findings suggest that the strategic combination of HA and probiotics may serve as a promising antibiotic‐alternative strategy in broiler production. However, the relatively short experimental period, modest sample size, and lack of in‐depth microbiome and immune function analyses limit broader conclusions. Therefore, further long‐term studies incorporating molecular microbiome profiling and detailed immunological assessments are recommended to clarify the underlying mechanisms and validate the applicability of these findings under commercial production systems.

## Funding

No funding was received for this study.

## Ethics Statement

The experimental protocol was approved by the Institutional Animal Ethics Committee of Sher‐e‐Bangla Agricultural University, Department of Animal Nutrition, Genetics, and Breeding (Ref: SAU/2023/ANGB‐04). The study was carried out in full compliance with ethical standards at the National Research and Development Poultry Farm, operated by National Hatchery Ltd., Dighulia, Tangail. All experimental procedures were conducted following the established guidelines for the ethical use and care of animals in research.

## Conflicts of Interest

The authors declare no conflicts of interest.

## Data Availability

The data that support the findings of this study are available from the corresponding author upon reasonable request.
